# Exploring potentialities of avian genomic research in Nepalese Himalayas

**DOI:** 10.1186/s40657-021-00290-5

**Published:** 2021-10-30

**Authors:** Prashant Ghimire, Nishma Dahal, Ajit K. Karna, Surendra Karki, Sangeet Lamichhaney

**Affiliations:** 1 Department of Biological Sciences, Kent State University, Kent, OH USA; 2 Biotechnology Division, CSIR-Institute of Himalayan Bioresource Technology, Palampur, HP India; 3 Center for Health and Disease Studies-Nepal, Kathmandu, Nepal; 4 Institute of Agriculture and Animal Sciences, Tribhuvan University, Kathmandu, Nepal; 5 Emergency Centre for Transboundary Animal Diseases, Food & Agricultural Organization of the UN, Kathmandu, Nepal; 6 School of Biomedical Sciences, Kent State University, Kent, OH USA

**Keywords:** Avian fauna, Evolutionary Process, Genomics, Himalayas, Nepal

## Abstract

Nepal, a small landlocked country in South Asia, holds about 800 km of Himalayan Mountain range including the Earth’s highest mountain. Within such a mountain range in the north and plain lowlands in the south, Nepal provides a habitat for about 9% of global avian fauna. However, this diversity is underrated because of the lack of enough studies, especially using molecular tools to quantify and understand the distribution patterns of diversity. In this study, we reviewed the studies in the last two decades (2000‒2019) that used molecular methods to study the biodiversity in Nepal to examine the ongoing research trend and focus. Although Nepalese Himalaya has many opportunities for cutting-edge molecular research, our results indicated that the rate of genetic/genomic studies is much slower compared to the regional trends. We found that genetic research in Nepal heavily relies on resources from international institutes and that too is mostly limited to research on species monitoring, distribution, and taxonomic validations. Local infrastructures to carry out cutting-edge genomic research in Nepal are still in their infancy and there is a strong need for support from national/international scientists, universities, and governmental agencies to expand such genomic infrastructures in Nepal. We particularly highlight avian fauna as a potential future study system in this region that can be an excellent resource to explore key biological questions such as understanding eco-physiology and molecular basis of organismal persistence to changing environment, evolutionary processes underlying divergence and speciation, or mechanisms of endemism and restrictive distribution of species.

**Supplementary Information:**

The online version contains supplementary material available at 10.1186/s40657-021-00290-5.

## Background

### Nepal and the Himalayas

**Fig. 1:**
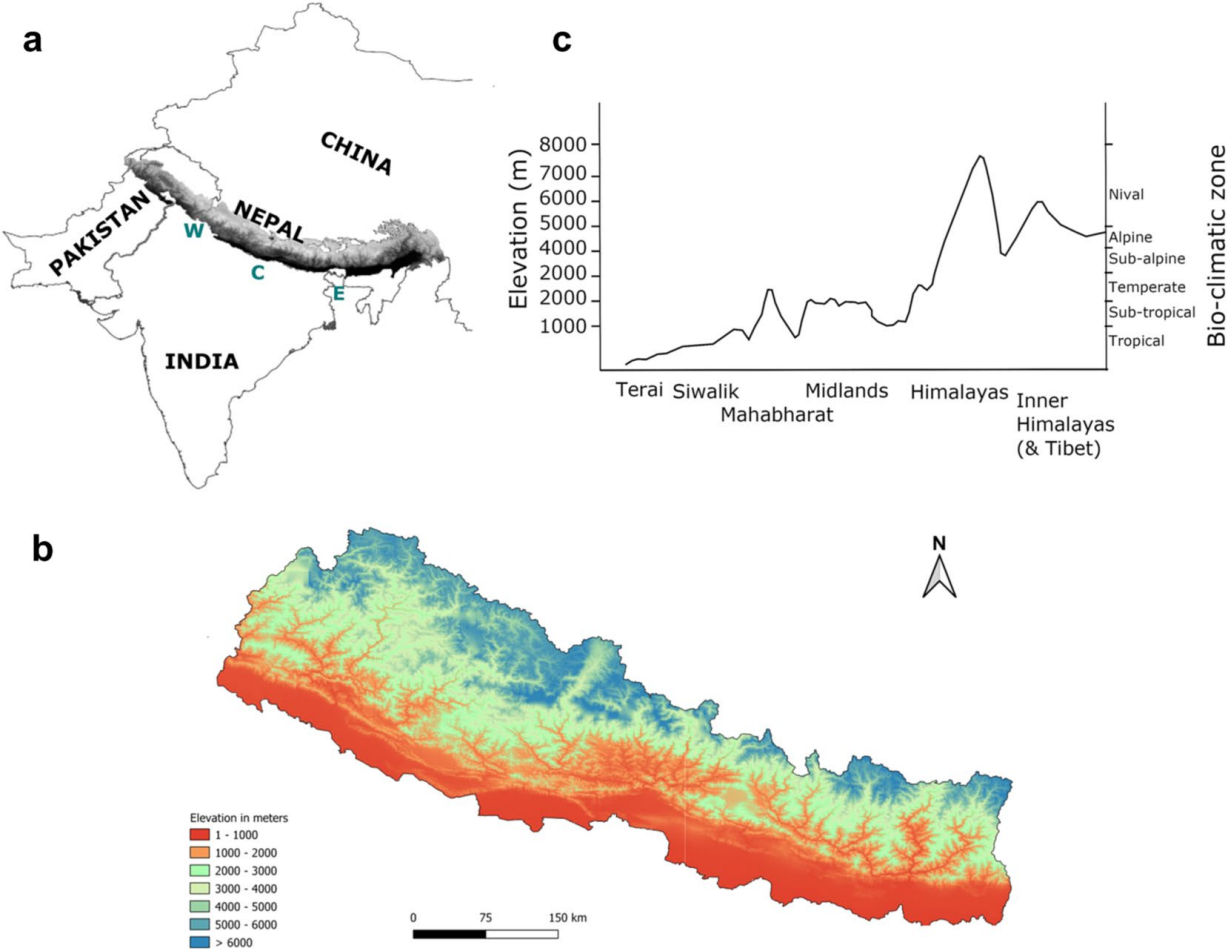
The Himalayas and Nepal. **a** Geographic range of the Himalayas that are divided into western (W), central (C), and eastern (E) zones. The eastern Himalayas extends from eastern Nepal across northeast India, Bhutan, Tibet to Yunnan in China and northern Myanmar. Western Himalayas refers to stretch from northeastern Afghanistan, southern Tajikistan, Pakistan through North India. In Nepal, eastern Himalayas starts around the Arun river followed by central and western demarcated around Kaligandaki gorge (Päckert et al. 2012). **b** Map of Nepal showing an altitudinal gradient of Nepalese Himalayas. **c** Bio-climatic and physiographic zones in Nepal, modified from Paudel et al. (2011)

Altitudinal gradients are characterized by steep changes in the physical environment (e.g. temperature, atmospheric oxygen level, ultraviolet (UV) radiation, geography) that present major and recurring challenges for successful organismal adaptation (Abbott and Brennan 2014). One such unique place on earth characterized by dramatic changes in the features of the altitudinal gradient is the Himalayas, which has the potential to serve as an ideal natural laboratory to study mechanisms of physiological adaptations and species assemblage. It stretches in an arc of 3000 km over northern Pakistan, China, Nepal, Bhutan, and the north-western and north-eastern states of India (Fig. 1a) and is home to some of the highest mountains in the world. The east and the west Himalayas also constitute a latitudinal gradient; the eastern Himalayas is placed at a lower latitude compared to the western Himalayas. These changes in altitudinal and latitudinal gradient has determined the climatic history of the Himalayas and perhaps shaped the current species diversity and richness patterns (Srinivasan et al. 2014). Ample evidence supports the Himalayas as a hotspot for biogeographic studies, but limited studies have investigated the biogeographic patterns of these montane species in the Himalayas (Rana et al. 2021).

Nepal, a small country of South Asia, has a diverse landscape geographically. There are plain basins in the south while the Himalayas stand up in the northern region of the country (Fig. 1a, b). Nepalese Himalaya constitutes an important part of the Himalayan range with 16% land area of the eastern Himalayas (Sharma et al. 2009) and the major part of western Himalayas (Fig. 1a). Within a mere 150 km north, there is a striking change in climate across Nepal i.e. the tropical zone to the nival zone with permanent frost and snow (Fig. 1c), that has led to the accumulation of rich biodiversity. Although Nepal occupies less than 0.1% of the global landmass, the country is known to be home to 2% of the flowering plants, 3% of the pteridophytes and 6% of the bryophytes, 3.9% of the mammals, 3.7% of the butterflies and 8.9% of the birds worldwide (Paudel et al. 2011).

Eight among ten tallest mountains in the world are found in Nepal including Mount Everest—the highest peak in the world. Life in a high-altitude environment brings numerous challenges posed by high-altitude stressors, relative to a low-altitude environment (Witt and Huerta-Sánchez 2019). With an increase in altitudes, the barometric and partial pressure of oxygen (PO_2_) is reduced, resulting in hypobaric hypoxia (Bouverot 1985). Within such hostility, to maintain an adequate supply of oxygen into the cells, high-altitude life requires short- and long-term physiological changes (Laguë 2017). Hence, the Nepalese landscape has a vast potential to provide natural laboratories to investigate mechanisms of physiological adaptation across the altitudinal gradient. The Himalayas are also considered a global biodiversity hotspot because the sharp rise in altitudinal gradient has resulted in a diverse ecosystem (Mittermeier et al. 2011). Hence, Nepal also provides ample opportunities for the studies of the patterns and mechanisms of genetic adaptation, species richness, and diversity along the altitudinal gradient.

Life in high altitudes is physiologically challenging for all animals, yet a wide variety of vertebrates can locally adapt in these orogenic regions, the most prolific being birds (Laguë 2017). Birds are known to possess more hypoxia tolerance compared to mammals because their oxygen transportation pathways have several unique features to support vigorous aerobic metabolism during hypoxia (Scott 2011). Due to these physiological adaptations, many bird species are endemic to high altitudes, and others are adapted across broad altitudinal gradients. Smaller and less complex genome size in birds compared to mammals makes them the popular model system to study physiological response to progressive hypoxia in high altitudes (Ivy et al. 2019; Laguë et al. 2020). Hence, we believe that studies of birds in the Nepalese landscapes will provide an excellent system to explore the patterns of biodiversity across the largest altitudinal gradient in the world and identify the underlying mechanisms of organismal persistence in a challenging environment.

Clues underlying specific evolutionary processes responsible for species diversity or adaptation are known to be hidden in the “genes” that control organismal phenotypes. Recent advancements in genomics allow us to use hypothesis-free screening of entire genomes to explore the biodiversity and adaptive potential in an organism at a molecular scale at different hierarchical levels (reviewed in Lamichhaney et al. 2019). However, due to systematic limitations including extreme climate, rugged mountainous terrain, and inaccessible habitat in addition to operational issues including lack of expertise, efforts to study species that have successfully adapted to the extreme conditions of the Himalayas have been challenging (Rana et al. 2021). Cutting-edge genomic methods such as next-generation sequencing are still underutilized in Nepal due to the lack of skilled human resources, appropriate technology, and fostering government policy (Basnet et al. 2019). In such context, this paper highlights the potentiality of genomics studies in Nepal with a particular focus on avian fauna. For this  review paper, we define genetic study as one that utilizes traditional methods (usage of mitochondria, few nuclear markers, or candidate genes), and genomics as the one that utilizes modern methods (high-throughput next-generation sequencing and bioinformatic analysis of large-scale data).

### Status on legislatures for carrying out genetic/genomic studies in Nepal

Legislatures regarding the genetic/genomic studies in Nepal are regulated by appropriate government authorities. For field studies within protected areas (i.e. national parks, conservation areas, wildlife reserves, hunting reserves, and buffer zones), prior approval from the Department of National Parks and Wildlife Conservation (DNPWC) under the Ministry of Forests and Environment (MoFE) is needed. Similarly, the Department of Forests and Soil Conservation (DoFSC) of MoFE is an authorized body to obtain approvals for studies to be carried outside the protected areas. “National Parks and Wildlife Conservation (NPWC) Act 1973” is the official act that guides the collection of specimens/samples for research. For most genetic studies undertaken in the past, researchers were requested to submit a proposal with all the necessary details on the study including the details of research facilities in Nepal where these genetic studies were carried out. The usage of study specimens and the genetic materials of the animal species beyond the objectives of the proposed research is strictly prohibited. In addition, for studying species that are enlisted in CITIES agreement, a letter of agreement from both exporting and importing parties is required (DFSC 2019). Furthermore, the local institutes conducting research with or without partnership with international institutes are also required to obtain approval from Social Welfare Council under Social Welfare Act, 1992.

**Fig. 2:**
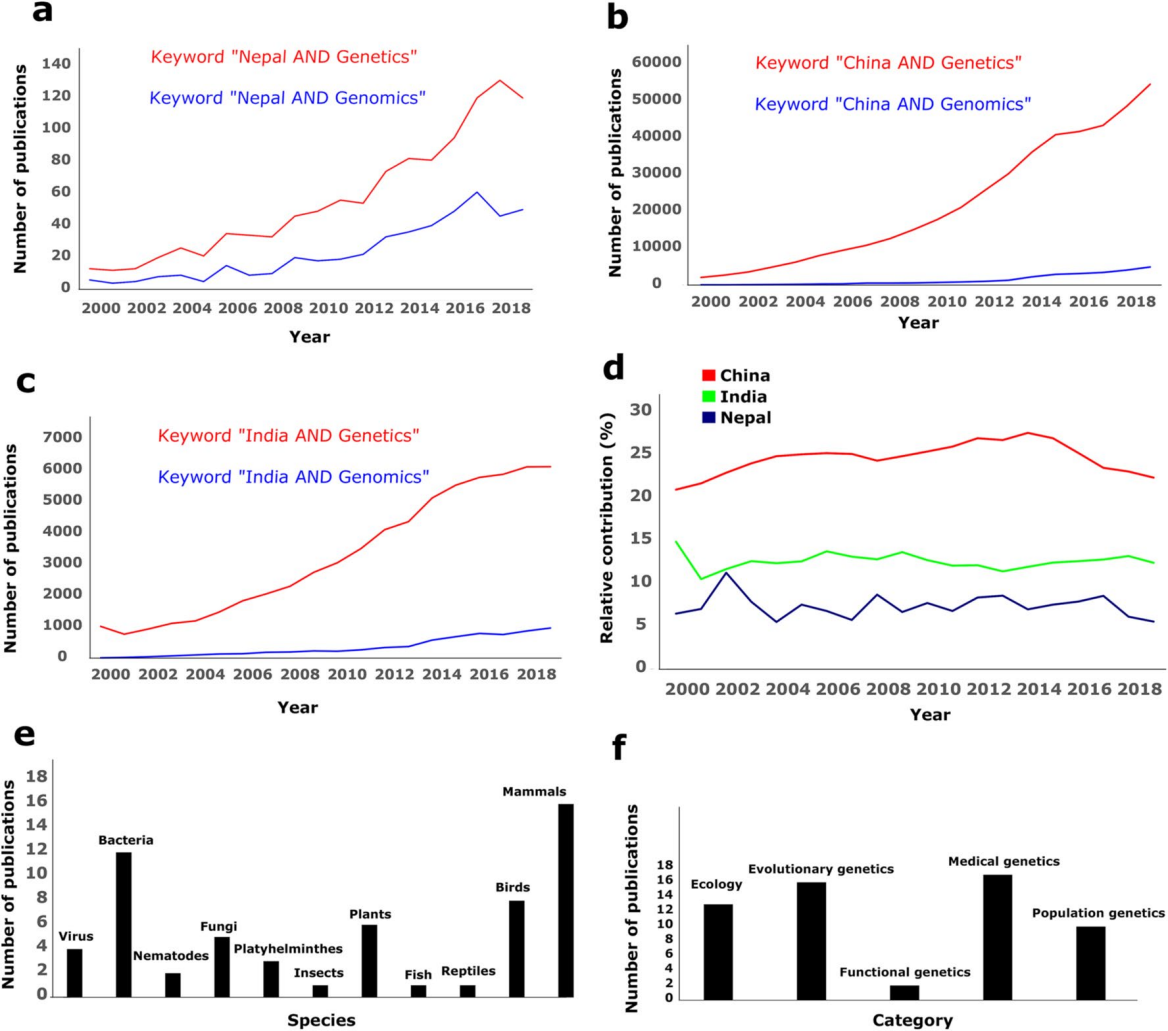
Current trend of genetic/genomic studies. **a** Trends of genetic/genomic research in Nepal in last two decades; **b** trends of genetic/genomic research in China in last two decades; **c** trends of genetic/genomic research in India in last two decades; **d** relative contribution of genetic/genomic research to overall life science research in Nepal, India, and China in last two decades. **e** Number of publications across different lineages among 59 studies which were exclusively carried out in Nepal (details in Additional file 1: Table S1) in last two decades; **f** number of publications across various fields of study carried out in Nepal

There are systematic complexities in conducting genetic/genomic research in Nepal. Legislatures regarding these studies in Nepal are very strict and complex, which perhaps is one of the important reasons that hinder genetic studies in Nepal (Fig. 2). It appears that related authorities in Nepal are still unaware of the true potential of genomic studies in Nepal. We hope a review paper like this will help stir the discussion about these issues to smoothen the legislative procedures for carrying out such studies in Nepal.

### Status of local infrastructures to carry out genetic/genomic research in Nepal

Nepal currently has 85 (public = 49, private = 36) molecular genetic laboratories; most of which were set up in recent times in response to the global COVID-19 pandemic for diagnostic purposes. In addition, government institutions such as Nepal Agricultural Research Council (NARC), Nepal Academy of Science and Technology (NAST), Central Veterinary Laboratory (CVL), Seed Quality Control Centre (SQCC), Department of Agriculture, different departments of Tribhuvan University, and Kathmandu University have genomics facilities to carry out such studies. Furthermore, the National Forensic Science Laboratory under the Ministry of Education, Science and Technology and Central Police Forensic Science Laboratory under Nepal Police also have genetic laboratories, particularly used for paternity testing and criminal identification. National Trust for Nature Conservation (NTNC) has also developed a molecular genetic laboratory at the Biodiversity and Conservation Center in Sauraha, Chitwan. In addition to these government institutes, private laboratories such as the Centre for Molecular Dynamics Nepal (CMDN), Kathmandu Center of Genomics and Research Laboratory (KCGRL), Intrepid Nepal Private Limited (INPL), Decode Genomics and Research Center, Centre for Health and Disease Studies (CHDS) and Research Institute for Bioscience and Biotechnology (RIBB) also have genomics facilities to conduct diagnostic as well as molecular biology research.

### Status of genetic/genomic studies in Nepal

We conducted PubMed literature searches on the date June 7, 2021, for topics “Genetics” + “Nepal”, and “Genomics” + “Nepal” to review published literature on genetic/genomic studies in Nepal. We only included original articles from the last two decades (2000–2019) to focus on contemporary trends. We did not use the records from 2020 as publications on that year might be affected by the global COVID-19 pandemic. The genetic/genomic studies conducted in Nepal (or utilizing samples from Nepal) have been in their lower end in the past but have been consistently on the rise (Fig. 2a). We compared this result with similar literature searches from two neighboring countries, China and India (Fig. 2b, c) to examine the regional trend. Although the number of genetic/genomic studies in China and India is significantly high (as indicated by the volume of yearly publications), the increasing usage of genetic/genomic methods in the last two decades is a common trend among these countries. However, the rate of increase of such studies in Nepal is much slower compared to China or India. We also examined the total number of publications in the field of life sciences from these three countries over the last two decades and estimated the relative contribution of genetic/genomic methods in these publications. On average, only ~ 7% of these publications from Nepal utilized the genetic/genomic approach, whereas the relative contributions in India (~ 13%) and China (~ 25%) were higher (Fig. 2d) in the last two decades. These overall trends highlight the need and potential of genetic/genomic studies in Nepal. This literature search also identified that genetic studies in Nepal were mostly limited to doing phylogenetic studies to examine taxonomic validations (Additional file 1: Table S1).

Among these publications, we selected 59 studies that were exclusively carried out in Nepal (Additional file 1: Table S1) for further analysis. Mammals were the most common study system among them, followed by bacteria (Fig. 2e). Most of the studies were conducted in lowlands i.e. Terai (*n* = 19), followed by Himalaya (*n* = 14) and mid-hills (*n* = 14). Genetic/genomic studies were mostly used in the field of medical science, followed by evolutionary biology while their usage in ecology and functional/system biology were low (Fig. 2f). Regarding birds, we found a total of 8 publications (Fig. 2e) that had performed genetic/genomic studies in 28 species, with groups of finches and warblers being the commonly studied systems (Additional file 1: Table S1).

Almost all (except three) studies were conducted in collaboration with international institutes. The first author in 57.6% of publications (*n* = 34) were international and the rest were Nepalese scientists. International scientists were corresponding authors in 83.05% of publications (*n* = 49) while on only 16.95% of publications (*n* = 10), Nepalese scientists contributed as corresponding author. The number of international authors accounts for 62.8% of total authors who contributed to the genetic/genomic research in Nepal. Only 16 (out of 59) studies were published in collaboration with an associated institute from the Government of Nepal. Twenty-seven publications had less than 5 authors while others were published by a larger team.

The above results indicate that genetic/genomic studies in Nepal have a heavy dependency on international institutes. In general, these international collaborations provide (i) lab equipment/infrastructure for doing such studies, as a local research laboratory in Nepal may not have enough financial resources to set up such infrastructures, and (ii) skilled manpower, as the majority of local researchers in Nepal are not well trained in doing laboratory activities or bioinformatic analysis of large datasets generated from such studies. The scope of local researchers is mostly limited to providing logistic assistance for collecting samples and carrying out field studies. Therefore, we believe that future research projects in Nepal should develop a dedicated program for training local researchers and provide them skills of international standards for carrying out high-quality research. In addition, there is also a dire need for change in policy from the Government of Nepal that will allow allocation of more research funds to the local research labs that will decrease the heavy dependency on international institutes for doing such studies in Nepal. The lack of government involvement in such studies is also of concern and demonstrates the strong need for increased outreach activities for such state-level participation in these studies.

This survey of published literature discussed above was done to explore the general trend on the studies of basic research in Nepal in comparison to the regional trend and explore challenges for conducting such studies in Nepal. Further, we will particularly focus on the status and opportunities of studying avian systems in Nepal, as the major focus of this paper is to explore the potentialities of future avian genomic research in Nepal.

### Status of ornithology and diversity of birds in Nepal

Brian Hodgson paved the way for Nepalese ornithology back in the nineteenth century as he was the first to collect the records of ~ 600 bird species from Nepal (Cocker and Inskipp 1988). After the dawn of democracy in the 1950s, the Government of Nepal prioritized wildlife conservation by establishing protected areas that pioneered bird conservation in Nepal. Featuring Himalayan Monal i.e. Danphe (*Lophophorus impejanus*) — the national bird of Nepal in postage stamps in early 1959 perhaps can be referred to as dawn on official state-level interest in avian conservation in Nepal. So far, 18 bird species have been featured in Nepal’s postage stamps. The first bird field guide of Nepal in the English language was published in 1976 (Fleming et al. 1976).

The contribution from international ornithologists such as Carol and Tim Inskipp nurtured modern-day ornithology in Nepal by publishing and advocating avian research and conservation. More recently, the publication of the first comprehensive avian database of Nepal (Inskipp et al. 2016) is considered a key resource for conducting avian research in Nepal. The Government of Nepal has also endorsed action plans for species such as vultures (DNPWC 2015), Bengal Florican (DNPWC 2016), pheasants (DNPWC and DFSC 2018), and owls (DNPWC and DFSC 2020) showing commitment for long term conservation of avian species.

**Fig. 3:**
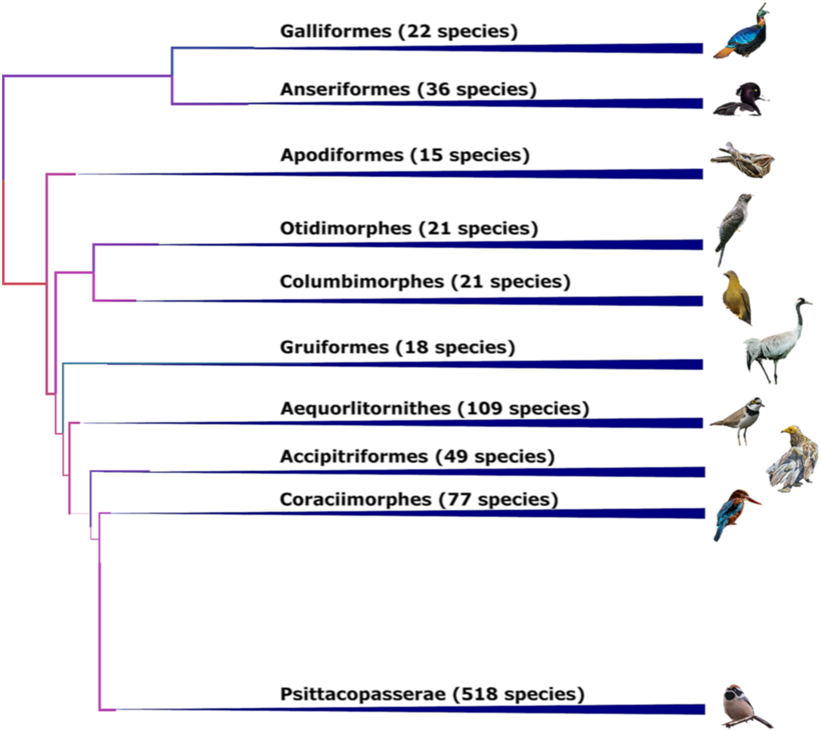
Phylogeny of birds in Nepal, tree modified from (Prum et al. 2015). The number in parenthesis indicates the total number of species in each branch that are found in Nepal. For details, see Additional file 1: Table S2. The birds shown are representative of each branch; Galliformes—Himalayan Monal (*Lophophorus impejanus*), Anseriformes—Tufted Duck (*Aythya fuligula*), Apodiformes—Large-tailed Nightjar (*Caprimulgus macrurus*), Otidimorphes—Common Cuckoo (*Cuculus canorus*), Columbimorphes—Yellow-footed Green-pigeon (*Treron phoenicoptera*), Gruiformes—Common Crane (*Grus Grus*), Aequorlitornithes—Little Ringed Plover (*Charadrius dubius*), Accipitriformes—Egyptian Vulture (*Neophron percnopterus*), Coraciimorphes—White-throated Kingfisher (*Halcyon smyrnensis*) and Psittacopasserae—Black-throated Tit (*Aegithalos concinnus*). The size of birds is not in relative scale and is only shown for demonstration. Bird images copyrights—Pemba Sherpa and Manshanta Ghimire

At present, Nepal holds 886 species of birds (DNPWC and BCN 2018). According to the most recent comprehensive phylogeny (Prum et al. 2015), birds in Nepal represent all major clades in the avian phylogeny (Fig. 3); representing 24 orders and 97 families (Additional file 2: Table S2). Passeriformes are the dominating order consisting of more than half of the bird species (499) followed by Charadriiformes (71) and Accipitriformes (49). Among these birds, 43 are globally threatened, 9 are protected, 1 is endemic, and 8 species are thought to be regionally extinct (Additional file 2: Table S2). Fifteen bird species are considered as restricted-range species as their range is restricted within a small area, i.e. species with an extent of occurrence less than 50,000 km^2^.

A general altitudinal trend observed about the species distribution around the globe is: regions closer to the tropics are species-rich because of the availability of diverse habitat (Brown 2014) whereas temperate high-altitudes are species-poor as they have large areas of similar habitat, resulting in species-specialized regions (Zou et al. 2016). A similar pattern is observed for avian fauna in Nepal; lowland regions of Nepal harbors a higher number of species while high mountains are species-poor (Baral and Inskipp 2020). About > 550 resident avian species undergo altitudinal migration (Inskipp et al. 2016), e.g. White-tailed Rubythroat (*Luscinia pectoralis*) and West Himalayan Bush Warbler (*Locustella kashmirensis*), which breed in the high Himalayas but migrate to lower altitude in winter (Baral and Inskipp 2020).

Past and ongoing ornithological studies have contributed to the understanding of the status, distribution, and ecology of the birds in Nepal (Baral et al. 2012). These studies are mostly led by several national non-government and local organizations in Nepal (Additional file 3: Table S3). These organizations are primarily focused on population monitoring, distribution, and understanding of the basic ecology of certain species like vultures, cranes, and owls.

### Previous avian genetic/genomic studies in Nepal

We find that most of the previous avian studies in Nepal that utilized genetic/genomic methods are molecular phylogenetic studies. While this is not due to discrepancy or lack of sufficient literature coverage for this review, this observation is consistent with the general research trend in Nepal (discussed above) on how genetic studies have been primarily used for taxonomic validations.

Studies on Nepalese avifauna have played important role in better understanding phylogenetic relationships especially through comparative studies. For example, Arnaiz-Villena et al. (2001) explored Nepalese avifauna and compared them with species found in other parts of the world. Mitochondrial cytochrome *b* (Cyt*b*) from nine species of goldfinch, bullfinches, grosbeaks, and rosefinches collected from Nepal were compared among 24 Carduelini species across the globe. A similar study sequenced Cyt*b* gene in 7 cryptic species of the Golden-spectacled Warbler (*Seicercus burkii* complex) from lowlands, mid-hills, and Himalayas of Nepal (Päckert et al. 2004) to reconstruct molecular phylogeny among these species and study intergeneric comparison between *Seicercus* and *Phylloscopus*. This study also identified low genetic diversity in higher altitude species in the Himalayas compared to the same species at the same altitudes of China, indicating at least partially restricted gene flow in these species. Gill et al. (2005) studied mitochondrial Cyt*b* gene in 40 species in the family Paridae that included museum specimen of 3 species of tits originally collected from Nepal. These studies also indicated that the Himalayas is one of the geographical regions across the globe with a higher rate of species diversification and endemism.

Recently, a phylogenetic study (Cibois et al. 2018) was carried out that included species endemic in Nepal, Spiny Babbler (*Turdoides nipalensis*). This study sequenced four nuclear introns from genes glyceraldehyde-3-phosphate dehydrogenase intron 11 (*GAPDH*), myoglobin intron 2 (*MYO*), ornithine decarboxylase introns 6 to 7 (*ODC*) and transforming growth factor beta 2 (*TGF-β2*), and one protein-coding mitochondrial gene, cytochrome oxidase subunit 1 (*CO1*). Their study suggested that Spiny Babbler is a close relative of White-throated Mountain Babbler (*Kupeornis gilberti*), an endemic of Cameroon and Nigeria, and Chapin’s Babbler (*Kupeornis chapini*), an endemic of Congo.

Similar phylogenetic studies were carried out that used a similar approach of mitochondrial and nuclear markers sequencing for taxonomic identifications and examining phylogeographic patterns, such as in Buff-barred Warbler (*Phylloscopus pulcher*; Päckert et al. 2014), Scaly-breasted Wren-babbler (*Pnoepyga albiventer*), and Nepal Wren Babbler (*Pnoepyga immaculata*; Päckert et al. 2013). Päckert et al. (2010) provided molecular phylogeny of Long-tailed Tits and allied species which include Black-throated Bushtit (*Aegithalos concinnus iredalei*), Rufous-fronted Bushtit (*Aegithalos iouschistos iouschisto*), and White-throated Bushtit (*Aegithalos niveogularis*) from Nepal.

All these studies discussed above highlight two common trends in genetic studies of avian fauna in Nepal: usage of the traditional approach of mitochondrial and a handful of nuclear markers that are being used mostly for phylogenetic studies and taxonomic identifications. There are no studies on avian fauna from Nepal to date that have harnessed the potential of the cutting-edge, high-resolution population and comparative genomic approaches to characterize the avian biodiversity in Nepal, and particularly the birds in the Himalayas to understand the molecular methods of high-altitude adaptation.

### Opportunities for future avian genomic studies in Nepal

We carried out a careful examination of published literature and unpublished data from local ornithologists (personal communications), and discussed with local experts and institutes to explore possible opportunities of future avian research in Nepal that can be benefitted by the usage of cutting-edge genomic tools. We also accounted for species spatial distribution, ecology, ease of sampling, and local logistics for doing field ornithological research while exploring these possible research themes.

As Nepal shares the Himalayan borders with other countries, birds in Nepal may also be distributed among other countries. Hence, there was the possibility that genomic studies in some of these proposed species may have been done in these countries, such as China and India where the usage of cutting-edge genomic methods in life science research is significantly higher than in Nepal (Fig. 2). So, in addition to the literature search using the “Nepal” keyword, the species proposed for carrying out future avian genomics studies in Nepal was also considered by taking into account if such comparative genomics work has already been done elsewhere in the world. We used the international public genome database (https://www.ncbi.nlm.nih.gov/genome/) to check if genomic resources of these proposed species are already generated elsewhere and only highlighted those species in this review that do not have any genomic records to date on such public genome databases. However, we acknowledge that there are other high-altitude systems around the world (e.g. Tibetan plateau, Andes) where similar studies (e.g. the genetic basis of high-altitude adaptations) have been done. Studies on similar biological questions in the Nepalese Himalayas will allow us to compare the findings and particularly examine if similar genetic mechanisms are associated with high altitude adaptations or if they are different for different mountain systems.

#### Collection of tissues or other materials

The protocol for the collection of a high-quality tissue sample is the key aspect of a successful genomic study. The future collection of tissues should follow the general global trend but take into consideration the recommendation of local authorities. The birds are captured with mist nets. The choice of the net would depend on the size of the target bird species. Nets are generally placed in the birds’ passage zone, installed in the evening or morning, and sampled immediately to avoid distress and injury to the birds. The birds are then placed into the bags or cage and non-invasive blood sampling is done at the point of capture and immediately released. Only trained researchers/assistants will be allowed for bird handling and sampling. The samples are temporarily stored in the cold chain box with its designed protocol of storing media and immediately transported to the molecular genetic laboratory. The long-term storage of the samples in the lab will be done at − 80 °C until they are further processed for genetic/genomic studies.

#### Studies of ecophysiology and molecular basis of high-altitude adaptation

**Fig. 4:**
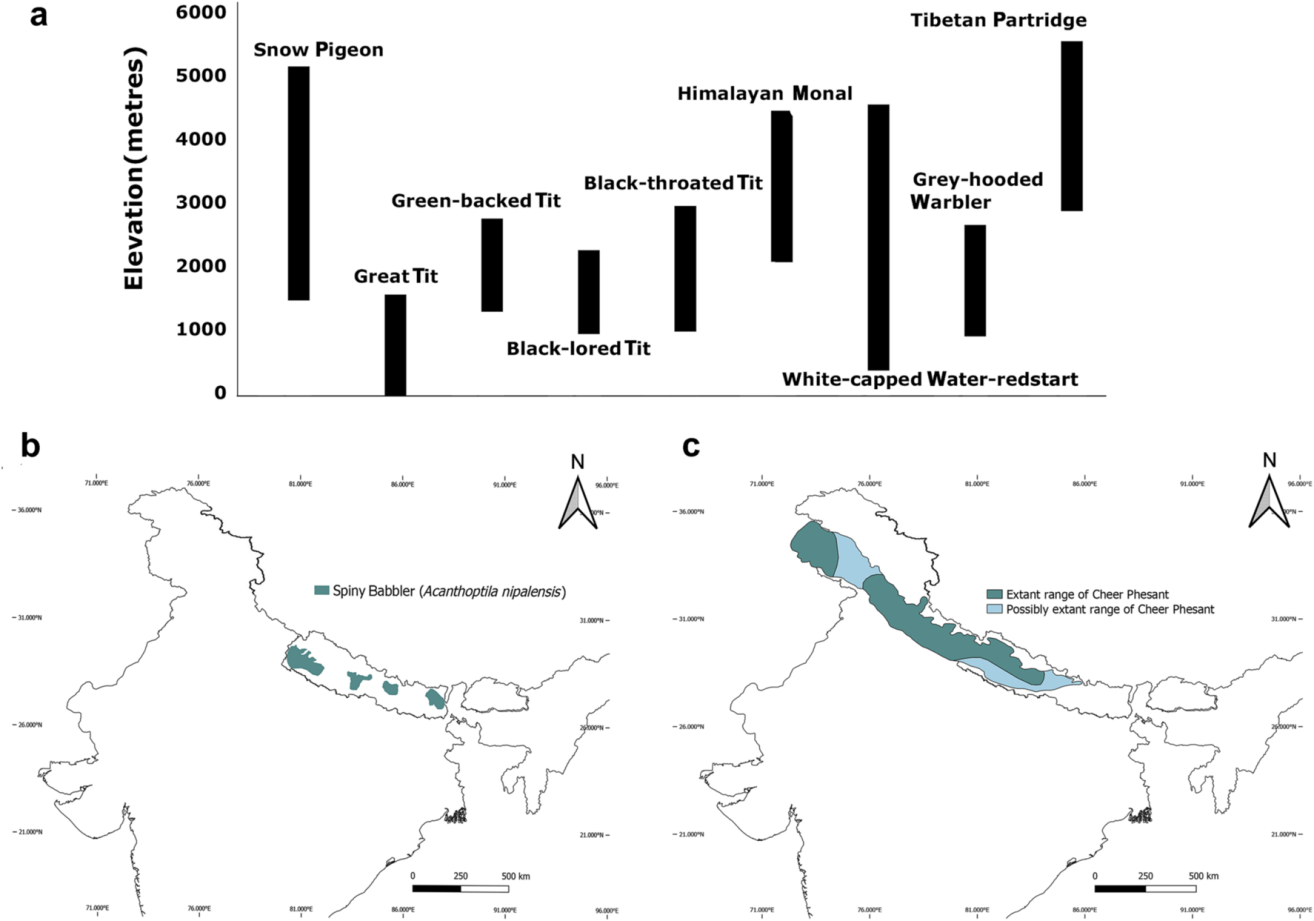
Some examples of future avian genomics research opportunities in Nepal. **a** Examples of species that are locally adapted across varying altitudes in the Himalayas. The lower and upper altitudinal limit of each species is shown. Data taken from Inskipp et al. (2016). **b** Habitat distribution of Spiny Babbler (*Acanthoptila nipalensis*), the species only found in Nepal and candidate for studies of endemism. **c** Habitat distribution of Cheer Pheasant (*Catreus wallichii*), a candidate species for studies of restrictive habitat distribution

Altitudinal gradients in the Nepalese Himalayas provide an ideal natural laboratory to explore the molecular basis of physiological acclimation and high-altitude adaptation. Many species are locally adapted across varying altitudes in the Himalayas (Fig. 4a) that can be used as study systems for such studies. One such species is the Snow Pigeon (*Columba leuconata*) that demonstrates extensive altitudinal migration, 1500 m in winter to 5200 m in summer (Inskipp et al. 2016). This species can be used to explore a fundamental biological question—whether phenotypic plasticity and genetic adaptation are structured and vary across a species’ habitat range, particularly in species that span dramatic altitudinal gradients. Moreover, an extensive amount of genomic studies have been done in one of its close relatives, Common Pigeon (*Columba livia*), which will aid in such comparative genomic studies (Bruders et al. 2020).

Another species that can be used for similar studies is Great Tit (*Parus major*), a popular study system in the field of evolutionary biology (Santure et al. 2013; Laine et al. 2016). They are found widespread in lowlands (~ up to 1600 m) (Inskipp et al. 2016). Its close relative, Green-backed Tit (*Parus monticolus*), is found in mid-hills (1300‒2800 m) and is known to perform altitudinal migration. Another close relative, Black-lored Tit (*Parus xanthogenys*), is resident from 900 to 2300 m but a rare visitor on lowlands (Inskipp et al. 2016). Black-throated Tit (*Aegithalos concinnus*) is another common tit found from 1000 to 3000 m altitude (Inskipp et al. 2016). These group of tits can be a useful model system to explore intra- and inter-species variation in patterns of genetic adaptation to altitudinal gradients.

#### Phylogenomics and studies of evolutionary processes underlying divergence and speciation

Ascertaining the phylogenetic position of a species together with its close relatives is one of the fundamental steps in the studies of biodiversity. The usage of genomics provides high-resolution molecular markers to explore underlying mechanisms of divergence and speciation in various avian species (Lamichhaney et al. 2016, 2018, 2020). High-resolution genomic markers generated from whole-genome sequencing methods can be used to explore phylogenomics and speciation in Himalayan Monal (*Lophophorus impejanus*) a species that is currently recognized as the national bird of Nepal. The Himalayan Monal is facing unusually high pressure from hunting and is therefore enlisted as near threatened species (Inskipp et al. 2016). It is a common resident pheasant in the central Himalayas with widespread distribution (high altitude resident, but migrating to lower altitudes during winter). Previous phylogenetic studies using mitochondrial sequences have conflicting results regarding the genetic relationship among different species in the family Phasianidae (Johnsgard 1978; Zhang et al. 2003; Chen et al. 2018). The taxonomic affinity of *Lophophora* itself is contradicted in previous studies (Huang et al. 2009; Shen et al. 2010, 2014). Most phylogenetic studies in the past have used a limited snapshot of the genome (limited mitochondrial or nuclear markers, microsatellites, etc.) which provide low-resolution data and, in many cases, examining full-scale genomic data improves resolution to study these phylogenetic relationships. Our previous studies have shown how usage of such full-scale genomic data has resolved phylogenetic conflicts in certain species (Lamichhaney et al. 2012, 2015). However, there are cases where even with the genome-scale data, the phylogenetic conflicts remain (see Wang et al. 2017; Kimball et al. 2021) and it requires a better understanding of the demographic and natural history of the study system to resolve such conflict.

The genome of one of the sister species of Himalayan Monal, Chinese Monal (*Lophophorus lhuysii*), has been published (Cui et al. 2019), that have found numerous expanded gene families and positively selected genes involved in high-altitude adaptation to low temperature and hypoxia. Hence, genome sequencing of Himalayan Monal would provide an opportunity to carry out comparative genomics studies with an already published genome of its sister species, Chinese Monal. Such studies would be important to understand the process of divergence between the two species and to explore the possibilities of convergent evolution of high-altitude adaptive traits among these species.

Similarly, Red-vented Bulbul (*Pycnonotus cafer*)—the most common bulbul species in Nepal (distributed from 75 to 2100 m altitude; Inskipp et al. 2016) could be another model system to study mechanisms of species divergence in the Himalayas by doing comparative genomic studies with one of its close relatives, Red-whiskered Bulbul (*Pycnonotus jocosus*), which is found only up to 350 m altitudes. Studies of introduced Red-whiskered Bulbul in Mauritius have identified rapid morphological divergence, particularly in bill size, in fewer than ten generations (Amiot et al. 2007). As Red-whiskered Bulbuls are resident and native to Nepal, there is an interesting opportunity to explore if rapid morphological divergence observed in introduced populations in Mauritius has also occurred in native populations in Nepal and study the genetic basis of such rapid morphological divergence.

#### Mechanisms of endemism and restrictive distribution of species

Endemism and restrictive distribution of species are often known as a consequence of survival in refugia (Tribsch and Schönswetter 2003). Few bird species in Nepal demonstrate such interesting restrictive distributions, potentially resulting from drastic species turnover due to late Pleistocene climate change (Dong et al. 2021). One such species is Spiny Babbler (*Turdoides nipalensi*)—the bird endemic to Nepal that shows the limited geographic distribution within Nepal too (Fig. 4b). Detailed genomic characterization of different populations of this species across Nepal will allow us to understand its demographic mechanisms associated with endemism and may provide important information for the future conservation efforts of this unique bird found in Nepal.

Cheer Pheasant (*Caterus wallichi*), a resident in the west of Kaligandaki river (Inskipp et al. 2016), is also a species showing restrictive habitat distribution (Fig. 4c). Interestingly, this species has never been recorded east of Kaligandaki. Kocklass Pheasant (*Pucrasia macrolopha nipalensis*) has an almost similar distribution as Cheer Pheasant with no record from the east while other pheasants such as Himalayan Monal, Blood Pheasant, Kalij Pheasant are widely recorded in the east. Genomic comparisons of these species will allow us to explore the molecular mechanisms associated with such restrictive habitats and local genetic adaptations. In addition to characterizing genomic pathways of local adaptation, mechanisms underlying biotic factors (e.g. species competition) and abiotic factors (e.g. geographic barriers) are also key for exploring such examples of endemism and restrictive distribution of species. Integration of genomic data with ecological and natural history data will allow us to study such biotic and abiotic factors associated with endemism and restrictive distribution of species.

#### An analytical framework for future genomic studies in Nepal

Setting up a genomic project particularly to explore underlying processes of adaptation and divergence requires a robust experimental design in terms of intra- and inter-species comparisons and appropriate sample size to obtain appropriate statistical power for reliable interpretations of the results. Most species we proposed above for future avian genomic studies in Nepal do not have previous genomic data available. Hence, the first step in most of these studies will be to generate *de-novo* genomes using an integrated approach of (a) high coverage second-generation sequencing (i.e. Illumina Novaseq), (b) third-generation sequencing (e.g. Nanopore), and (c) three-dimensional (3D) organizational structure of chromatin (e.g. HiC) to produce chromosomal-scale reference genomes that can be used for downstream analysis.

Once the genomes of the reference species are generated, low coverage sequencing of multiple populations (within species) can be done and allow for carrying out a population genomics approach to study within-species divergence and local adaptation of a particular population in a specific environment. For the study of between-species comparisons, a comparative genomics approach can be done. Large-scale dataset on genetic variants (SNPs, indels, structural variants) identified using population genomics approach, e.g. pipeline used in Lamichhaney et al. (2015) or data on conserved genomic elements and regulatory regions identified using comparative genomics approach, e.g. pipeline used in Sackton et al. (2019) can be further used for genomic characterization of such underlying processes of adaptation and divergence.

## Conclusion

Nepal constitutes an important part of the Himalayas that has been considered a hotspot for biodiversity. Striking changes in altitudes within a short geographic distance has led to the accumulation of rich biodiversity that provides ample opportunities for the studies of patterns and mechanisms of species richness and diversity along the altitudinal gradient. In the current era, many biodiversity hotspots around the globe are richly benefiting from the usage of cutting-edge genomic tools to explore the patterns of biodiversity and study molecular mechanisms of organismal persistence in a novel environment. These studies are key to understanding the impacts of global and local climate change (Dahal et al. 2021). But our review of published literature indicated that only limited studies have been done in the Nepalese Himalayas that have utilized such a cutting-edge genomic approach to explore mechanisms of biodiversity and adaptation. Related studies done in Nepal have mostly used traditional genetic methods but that also has been mostly led by international institutions. The lagging genomic research in this region can be explained by (i) lack of well-equipped genomic facilities to set up a high-quality research program, (ii) restrictive governmental policies for utilizing the biological specimen for doing such research, and (iii) logistic challenges due to difficult geographic terrains. To increase the potentiality of future studies, we believe three important sectors need to be addressed: (i) improving infrastructures for genomic research in Nepal; the local laboratory in Nepal needs to be strengthened by installing genome sequencing instruments and associated infrastructures for carrying out cutting-edge genomic research. We believe, such genomic infrastructures should be built via government leadership that will further encourage other private institutions to come aboard; (ii) training and improving the skill set of local researchers; they should be exposed to genomic laboratories of international standards and trained to improve their laboratory and computational skills for analyzing large-scale genomic data. In addition, the inclusion of new courses in genomics and bioinformatics into the existing university course curriculum in Nepal will be an excellent way of developing next-generation scientists in the field of genomics; (iii) revision of legislatures procedures for getting permits on carrying out such studies. There needs to be flexibility in terms of legislative requirements depending on the nature and scope of genomic studies being proposed. In addition, the administrative delay in processing permit applications should be improved to provide a quicker response to the researchers as time is always a key factor for doing such cutting-edge research in the current era.

Having one of the most diverse habitats in the world from the snow-capped Himalayas to fertile plains, Nepal’s potentiality in the genomic study is underrated. Looking forward, the local research institutes and infrastructure should be strengthened in terms of human resources, technical expertise, and facilities to set up globally competing high-quality long-term research programs. Although any flora or fauna in the region can be developed as a model study system, we particularly highlight avian systems that can provide rich opportunities to achieve the above-mentioned objectives. After examination of published literature, analysis of unpublished data from local ornithologists, and discussion with local institutes, we have identified key research themes on how avian biodiversity in Nepal can be used to answer some of the fundamental biological questions, such as studies of ecophysiology and molecular basis of high-altitude adaptation, phylogenomics, and studies of evolutionary processes underlying divergence and speciation or mechanisms of endemism and restrictive distribution of species. One particular study we have already started in this region is to examine the relative roles of phenotypic plasticity and genetic adaptation in either facilitating or hindering organismal persistence to high altitude environments by integrating multi-omics approaches and field-based experimental studies.

## Supplementary Information

**Additional file 1: Table S1.** Details on the publications reviewed in this study.

**Additional file 2: Table S2.** List of birds found in Nepal.

**Additional file 3: Table S3.** Major organizations working on birds in Nepal.

## Data Availability

All the data generated and analyzed for this study can be found in supplementary materials.

## References

[CR1] Abbott RJ, Brennan AC (2014). Altitudinal gradients, plant hybrid zones and evolutionary novelty. Philos Trans R Soc Lond B Biol Sci.

[CR2] Amiot C, Lorvelec O, Mandon-Dalger I, Sardella A, Lequilliec P, Clergeau P (2007). Rapid morphological divergence of introduced Red-whiskered Bulbuls *Pycnonotus jocosus* in contrasting environments. Ibis.

[CR3] Arnaiz-Villena A, Guillén J, Ruiz-del-Valle V, Lowy E, Zamora J, Varela P (2001). Phylogeography of crossbills, bullfinches, grosbeaks, and rosefinches. Cell Mol Life Sci.

[CR4] Baral HS, Inskipp C, Regmi GR, Huettmann F (2020). Birds of Nepal: their status and conservation especially with regards to watershed perspectives. Hindu Kush—Himalaya watersheds downhill: landscape ecology and conservation perspective.

[CR900] Baral H, Regmi U, Poudyal L, Acharya R. Status and Conservation of Birds in Nepal. Biodiversity conservation in Nepal: a success story; 2012. p. 69–90.

[CR5] Basnet D, Kandel P, Chettri N, Yang Y, Lodhi M, Htun N (2019). Biodiversity research trends and gaps from the confluence of three global biodiversity hotspots in the Far-Eastern Himalaya. Int J Ecol.

[CR6] Bouverot P (1985). Adaptation to altitude-hypoxia in vertebrates.

[CR7] Brown JH (2014). Why are there so many species in the tropics?. J Biogeogr.

[CR8] Bruders R, Van Hollebeke H, Osborne EJ, Kronenberg Z, Maclary E, Yandell M (2020). A copy number variant is associated with a spectrum of pigmentation patterns in the rock pigeon (*Columba livia*). PLoS Genet..

[CR9] Chen Y, Li F, Zhang Q, Wang Q (2018). Complete mitochondrial genome of the Himalayan Monal *Lophophorus impejanus* (Phasianidae), with phylogenetic implication. Conserv Genet Resour.

[CR10] Cibois A, Gelang M, Alström P, Pasquet E, Fjeldså J, Ericson PGP (2018). Comprehensive phylogeny of the laughingthrushes and allies (Aves, Leiothrichidae) and a proposal for a revised taxonomy. Zool Scr.

[CR11] Cocker PM, Inskipp C (1988). A Himalayan ornithologist: the life and work of Brian Houghton Hodgson.

[CR12] Cui K, Li W, James JG, Peng C, Jin J, Yan C (2019). The first draft genome of *Lophophorus*: a step forward for Phasianidae genomic diversity and conservation. Genomics.

[CR13] Dahal N, Lamichhaney S, Kumar S (2021). Climate change impacts on Himalayan biodiversity: evidence-based perception and current approaches to evaluate threats under climate change. J Indian Inst Sci.

[CR14] DFSC. CITIES convention and related laws in Nepal. Babarmahal, Kathmandu: Department of Forest and Soil Conservation; 2019.

[CR15] DNPWC. Vulture conservation action plan for Nepal (2015‒2019). Department of National Parks and Wildlife Conservation. Kathmandu: Ministry of Forests and Soil Conservation, Government of Nepal; 2015.

[CR16] DNPWC. Bengal florican conservation action plan. Babarmahal: Department of National Parks and Wildlife Conservation; 2016.

[CR17] DNPWC, BCN. Birds of Nepal: an official checklist. Kathmandu: Department of National Parks and Wildlife Conservation; 2018.

[CR18] DNPWC, DFSC. Pheasant conservation action plan for Nepal (2019‒2023). Kathmandu: Department of National Parks and Wildlife Conservation and Department of Forests and Soil Conservation; 2018.

[CR19] DNPWC, DFSC. Owl conservation action plan for Nepal 2020‒2029. Kathmandu, Nepal: Department of National Parks and Wildlife Conservation and Department of Forests and Soil Conservation; 2020.

[CR20] Dong F, Hung CM, Li SH, Yang XJ (2021). Potential Himalayan community turnover through the Late Pleistocene. Clim Change.

[CR22] Fleming RL Sr, Fleming RL Jr, Bangdel LS. Birds of Nepal. Flemings Sr and Jr.; 1976.

[CR23] Gill FB, Slikas B, Sheldon FH (2005). Phylogeny of Titmice (Paridae): II. Species relationships based on sequences of the mitochondrial cytochrome-B gene. Auk..

[CR24] GON. National Parks and Wildlife Conservation Regulation (Fifth amendment). Government of Nepal; 2019.

[CR26] Huang Z, Liu N, Xiao Y, Cheng Y, Mei W, Wen L (2009). Phylogenetic relationships of four endemic genera of the Phasianidae in China based on mitochondrial DNA control-region genes. Mol Phylogenet Evol.

[CR27] Inskipp C, Baral HS, Phuyal S, Bhatt TR, Khatiwada M, Inskipp T (2016). The status of Nepal’s Birds: the national red list series.

[CR28] Ivy CM, Lague SL, York JM, Chua BA, Alza L, Cheek R (2019). Control of breathing and respiratory gas exchange in high-altitude ducks native to the Andes. J Exp Biol..

[CR29] Johnsgard P. Review of the pheasants of the world. 1978. https://digitalcommons.unl.edu/biosciornithology/79.

[CR30] Kimball RT, Hosner PA, Braun EL (2021). A phylogenomic supermatrix of Galliformes (Landfowl) reveals biased branch lengths. Mol Phylogenet Evol..

[CR31] Laguë SL (2017). High-altitude champions: birds that live and migrate at altitude. J Appl Physiol.

[CR32] Laguë SL, Ivy CM, York JM, Chua BA, Alza L, Cheek R (2020). Cardiovascular responses to progressive hypoxia in ducks native to high altitude in the Andes. J Exp Biol..

[CR33] Laine VN, Gossmann TI, Schachtschneider KM, Garroway CJ, Madsen O, Verhoeven KJF (2016). Evolutionary signals of selection on cognition from the great tit genome and methylome. Nat Commun.

[CR34] Lamichhaney S, Barrio MA, Rafati N, Sundström G, Rubin CJ, Gilbert ER (2012). Population-scale sequencing reveals genetic differentiation due to local adaptation in Atlantic herring. P Natl Acad Sci USA.

[CR35] Lamichhaney S, Berglund J, Almén MS, Maqbool K, Grabherr M, Martinez-Barrio A (2015). Evolution of Darwin’s finches and their beaks revealed by genome sequencing. Nature.

[CR36] Lamichhaney S, Fan G, Widemo F, Gunnarsson U, Thalmann DS, Hoeppner MP (2016). Structural genomic changes underlie alternative reproductive strategies in the ruff (*Philomachus pugnax*). Nat Genet.

[CR37] Lamichhaney S, Han F, Webster MT, Andersson L, Grant BR, Grant PR (2018). Rapid hybrid speciation in Darwin’s finches. Science.

[CR38] Lamichhaney S, Card DC, Grayson P, Tonini JFR, Bravo GA, Näpflin K (2019). Integrating natural history collections and comparative genomics to study the genetic architecture of convergent evolution. Phil Trans R Soc B Biol Sci.

[CR39] Lamichhaney S, Han F, Webster MT, Grant BR, Grant PR, Andersson L (2020). Female-biased gene flow between two species of Darwin’s finches. Nat Ecol Evol.

[CR40] Mittermeier RA, Turner WR, Larsen F, Brooks TM, Gascon C, Zachos F, Habel J (2011). Global biodiversity conservation: the critical role of hotspots. Biodiversity hotspots.

[CR41] Päckert M, Martens J, Sun Y-H, Veith M (2004). The radiation of the *Seicercus burkii* complex and its congeners (Aves: Sylviidae): molecular genetics and bioacoustics. Org Divers Evol.

[CR42] Päckert M, Martens J, Sun Y-H (2010). Phylogeny of long-tailed tits and allies is inferred from mitochondrial and nuclear markers (Aves: Passeriformes, Aegithalidae). Mol Phylogenet Evol.

[CR43] Päckert M, Martens J, Sun Y-H, Severinghaus LL, Nazarenko AA, Ting J (2012). Horizontal and elevational phylogeographic patterns of Himalayan and Southeast Asian forest passerines (Aves: Passeriformes). J Biogeogr.

[CR44] Päckert M, Martens J, Liang W, Hsu Y-C, Sun Y-H (2013). Molecular genetic and bioacoustic differentiation of *Pnoepyga* Wren-babblers. J Ornithol.

[CR45] Päckert M, Sun Y-H, Fischer BS, Tietze DT, Martens J (2014). A phylogeographic break and bioacoustic intraspecific differentiation in the Buff-barred Warbler (*Phylloscopus pulcher*) (Aves: Passeriformes, Phylloscopidae). Avian Res.

[CR47] Paudel PK, Bhattarai BP, Kindlmann P, Kindlmann P (2011). An overview of the biodiversity in Nepal. Himalayan biodiversity in the changing world.

[CR48] Prum RO, Berv JS, Dornburg A, Field DJ, Townsend JP, Lemmon EM (2015). A comprehensive phylogeny of birds (Aves) using targeted next-generation DNA sequencing. Nature.

[CR49] Rana SK, Rawal RS, Dangwal B, Bhatt ID, Price TD (2021). 200 years of research on Himalayan biodiversity: trends, gaps, and policy implications. Front Ecol Evol..

[CR50] Sackton TB, Grayson P, Cloutier A, Hu Z, Liu JS, Wheeler NE (2019). Convergent regulatory evolution and loss of flight in paleognathous birds. Science.

[CR51] Santure AW, Cauwer ID, Robinson MR, Poissant J, Sheldon BC, Slate J (2013). Genomic dissection of variation in clutch size and egg mass in a wild great tit (*Parus major*) population. Mol Ecol.

[CR52] Scott GR (2011). Elevated performance: the unique physiology of birds that fly at high altitudes. J Exp Biol.

[CR53] Sharma E, Chettri N, Tshe-ring K, Shrestha A, Jing F, Mool P, et al. Climate change impacts and vulnerability in the Eastern Himalayas. 2009. http://www.environmentportal.in/content/294619/climate-change-impacts-and-vulnerability-in-the-eastern-himalayas/.

[CR54] Shen Y-Y, Liang L, Sun Y-B, Yue B-S, Yang X-J, Murphy RW (2010). A mitogenomic perspective on the ancient, rapid radiation in the Galliformes with an emphasis on the Phasianidae. BMC Evol Biol.

[CR55] Shen Y-Y, Dai K, Cao X, Murphy RW, Shen X-J, Zhang Y-P (2014). The updated phylogenies of the Phasianidae based on combined data of nuclear and mitochondrial DNA. PLoS ONE..

[CR57] Srinivasan U, Tamma K, Ramakrishnan U (2014). Past climate and species ecology drive nested species richness patterns along an east-west axis in the Himalaya. Global Ecol Biogeogr.

[CR58] Thapa K, Manandhar S, Bista M, Shakya J, Sah G, Dhakal M (2018). Assessment of genetic diversity, population structure, and gene flow of tigers (*Panthera tigris tigris*) across Nepal’s Terai Arc Landscape. PLoS ONE..

[CR59] Tribsch A, Schönswetter P (2003). Patterns of endemism and comparative phylogeography confirm palaeo-environmental evidence for *Pleistocene refugia* in the Eastern Alps. Taxon.

[CR60] Wang N, Hosner PA, Liang B, Braun EL, Kimball RT (2017). Historical relationships of three enigmatic phasianid genera (Aves: Galliformes) inferred using phylogenomic and mitogenomic data. Mol Phylogenet Evol.

[CR62] Witt KE, Huerta-Sánchez E (2019). Convergent evolution in human and domesticate adaptation to high-altitude environments. Phil Trans R Soc B Biol Sci.

[CR63] Zhang ZW, Ding CQ, Ding P, Zheng GM (2003). The current status and a conservation strategy for species of Galliformes in China. Biodivers Sci.

[CR64] Zou Y, Sang W, Hausmann A, Axmacher JC (2016). High phylogenetic diversity is preserved in species-poor high-elevation temperate moth assemblages. Sci Rep.

